# Multi-Sensor Data Fusion for Remaining Useful Life Prediction of Machining Tools by IABC-BPNN in Dry Milling Operations

**DOI:** 10.3390/s20174657

**Published:** 2020-08-19

**Authors:** Min Liu, Xifan Yao, Jianming Zhang, Wocheng Chen, Xuan Jing, Kesai Wang

**Affiliations:** School of Mechanical and Automotive Engineering, South China University of Technology, Guangzhou 510640, China; melmliumr@mail.scut.edu.cn (M.L.); mejmingz@mail.scut.edu.cn (J.Z.); 201821002843@mail.scut.edu.cn (W.C.); 201710100411@mail.scut.edu.cn (X.J.); msx2jqkesai@mail.scut.edu.cn (K.W.)

**Keywords:** remaining useful life, machining tools, multi-sensor, data fusion, back propagation neural network, artificial bee colony

## Abstract

Inefficient remaining useful life (RUL) estimation may cause unpredictable failures and unscheduled maintenance of machining tools. Multi-sensor data fusion will improve the RUL prediction reliability by fusing more sensor information related to the machining process of tools. In this paper, a multi-sensor data fusion system for online RUL prediction of machining tools is proposed. The system integrates multi-sensor signal collection, signal preprocess by a complementary ensemble empirical mode decomposition, feature extraction in time domain, frequency domain and time-frequency domain by such methods as statistical analysis, power spectrum density analysis and Hilbert-Huang transform, feature selection by a Light Gradient Boosting Machine method, feature fusion by a tool wear prediction model based on back propagation neural network optimized by improved artificial bee colony (IABC-BPNN) algorithm, and the online RUL prediction model by a polynomial curve fitting method. An example is used to verify whether if the prediction performance of the proposed system is stable and reliable, and the results show that it is superior to its rivals.

## 1. Introduction

In an automatic manufacturing system, machining tools of computer numerical control (CNC) have always been a crucial factor for machining quality. Machining tools wear or breakage may significantly decrease machining quality, increase production costs or even interrupt the running of the manufacturing system [1], and it is estimated 20% of downtime is attributed to tool failures [2]. Therefore, online remaining useful life (RUL) prediction and replacement of machining tools in time are urgently needed to assure machining quality and system reliability [3].

A huge amount of research work on RUL prediction of machining tools or equipment has been done over the last decade. In general, RUL prediction methods are divided into main three kinds, which are experience-based models, physics-based models and data-based methods [4,5]. 

According to the observed situation, experience-based models usually utilize engineering experience and expert knowledge to infer RUL from historical data. In diagnostics and prognostics, fuzzy logic methods and expert systems are two typical experience-based methods. Khelif et al. proposed an experience-based method, which uses the experience gained from solving similar and already seen problems, to predict the RUL of Li-ion batteries [6]. Yan et al. presented a fuzzy logic combined logistic regression method to predict RUL of gas turbine hot components and to assess fatigue severity. The fuzzy logic of the method was derived by using engineers’ experience and historical maintenance running records [7]. Although, in many fields, it is a good solution to predict the RUL of equipment, there are some problems, for example, domain knowledge is relied upon heavily, system rules are difficult to define and fuzzy sets of system characteristics are difficult to develop. 

Utilizing physical mechanisms (e.g., abrasion, diffusion, the Wiener process) or mathematical models (e.g., regression, the Taylor formula) and the measured data, physics-based models can describe the degradation progress to estimate RUL. In diagnostics and prognostics, the model parameters are identified and updated by using in-process data and statistical methods. Baraldi et al. built a Monte Carlo-based filtering technique (a physics-based model), which is based on an observation equation to describe the relation between the system degradation states and the observation values, to predict the distribution of the system RUL and to update the online-observation data [8]. According to studies of the physical characteristic of cutting tool’s flank wear processes, Pálmai proposed a complex wear equation (a mathematical model) to calculate the tool life, to determine the Taylor formula of any tool life criterion and to optimize the technological process [9]. However, a physics-based model is often built case by case, requiring extensive experiments to acquire the model parameters and empirical data. In addition, it may not be suitable for complex systems.

Relying only on the data from on-line or history, data-based models can predict a system’s state or match similar historical patterns to infer RUL. There are some common data fusion models such as statistical models, artificial intelligence models and reliability functions. Statistical models, such as the discrete Bayesian filter, have been used to estimate the degradation state, and on this basis, a two phase data fusion method is presented for RUL prediction [10]. An improved Hidden Markov model (HMM) has been constructed to describe the time varying and condition adaptive state transition probability and to estimate on-line tool wear state and predict the tool RUL, whose hidden layer describes the process of wear, while the observation layer describes the relationship between tool wear state and sensing signals [11]. Artificial intelligence models, such as neural networks, support vector machines (SVM) and neuro-fuzzy inference system (NFIS) are often employed to model and estimate equipment status. Taking the unprocessed and preprocessed data as the input data, a multilayer perceptron neural network is selected for training to estimate the RUL of rolling element bearings [12]. Patil et al. proposed a novel method to real-time estimate the RUL of Li-ion batteries, which is based on classification and regression attributes of SVM. Using SVM and critical features, the classification and regression models for RUL are built and can predict multiple batteries accurately [13]. Razavi et al. proposed an adaptive NFIS to predict the RUL of aircraft engines by studying the degradation process of the engine with only the provided historical data [14]. Reliability functions such as Weibull distribution have been used to construct a Weibull accelerated failure time regression (WAFTR) model by Kundu et al. [15]. In the WAFTR model, the best principal component value and working condition, like load and speed, are used for predicting the RUL of the rolling element bearings. Therefore, data fusion models are suitable for systems where the data are sufficiently abundant, without understanding the complex physics. In prognostics applications, most of data fusion models are not easy to explain in physical meaning, even in determining thresholds and solving over-fitting issues [4].

From the literature on RUL, there is no universally accepted best RUL predication model, and each model has its advantages and disadvantages. As we all know, with the development of sensing technology, more and more various sensors such as acoustic emission, vibration and force variation are used in the condition monitoring of tools, and a large volume of machining process data is easy to obtain. Additionally, there are the complexity of failure mechanisms and the uncertainty of the model parameters of machining tools in cutting process [16], and the highly non-linear relationship between the obtained signals and tool wear condition make analysis or tool wear recognition using traditional methods very difficult [3]. For the above reasons, data fusion models are much easier to implement than the other two models in the RUL prediction of machining tools.

In recent years, a lot of the literature has been produced on data fusion models for the RUL prediction of machining tools [3,16,17,18,19,20,21,22,23], among which the acquisition of data mainly comes from sensing signals, including single sensor signal and multi-sensor signals. Compared to the single sensor signal, multi-sensor signals can provide more information about machining tools in machining process and make the RUL prediction result more reliable [3]. Thus, acquiring the most effective feature information and fusion from multi-sensor signals is a hot topic. Yu et al. proposed a novel weighted HMM-based approach for RUL prediction. The wear evolution process was discretized into five wear stages, and was formulated by multiple HMMs with different steps in each stage. The weighted HMM model was effectively fused based on multi-sensor signals and the predicted the RUL of tools [22]. Traditionally, feature extraction and selection is the key to multi-sensor data fusion. Many effective methods, like statistical analysis, time-frequency analysis and deep learning, have been used to extract features, and those, like correlation analysis, monotonicity analysis and residual analysis, have been used to select optimum features. Wu et al. utilized ensemble empirical mode decomposition method to eliminate noises of multi-sensor signals, statistic methods to extract feature, three methods including correlation analysis, monotonicity analysis and residual analysis to select optimum features, and adaptive NFIS to fuse feature, and then built an RUL prediction model [16]. Generally, soft computing techniques are applied for undertaking the fusion combing with some classical methods like SVM, NFIS and logistic regression, and an effective RUL of machining tool prediction model is ultimately formed. However, in the actual machining process, due to the randomness or nonlinearity between the level of tool wear and the feature of multi-sensor signals extracted and selected, the prediction model makes it difficult to predict the RUL of machining tools accurately and quickly.

In order to solve the above problems, an online RUL of machining tool prediction system, using back propagation neural network optimized by improved artificial bee colony algorithm (IABC-BPNN), based on multi-sensor data fusion is proposed in this paper. First, a multi-sensor data fusion online RUL prediction system scheme is introduced, which is based on massive sensor signals, and divided into an online signal data process and an offline signal data process. Then, the captured signals from force and vibration sensors are de-noised by a complementary ensemble empirical mode decomposition (CEEMD). The de-noised signals are used for effective feature extraction by statistical analysis, time domain analysis, frequency domain analysis and Hilbert-Huang transform (HHT). Next, a Light Gradient Boosting Machine (LightGBM) method-based feature selection is presented to obtain the optimal features. Finally, IABC-BPNN model is constructed to implement the feature fusion and predict the tool wear, and a polynomial curve fitting method (PCF) is used to predict online RUL of the machining tool.

The remainder of this paper is organized as follows: Section 2 proposes a RUL prediction system of machining tools based on multi-sensor data fusion. Section 3 introduces the signal preprocess method called CEEMD. Section 4 discusses different feature extraction methods in different domains, and the optimal features selection by the LightGBM method. Section 5 explains the IABC-BPNN prediction model-based data fusion and an online RUL prediction model building. Section 6 represents an experimental example study of the multi-sensor data fusion system, and discusses the experimental results. Section 7 summarizes the paper and looks forward to the future.

## 2. RUL Prediction System of Machining Tools Based on Multi-Sensor Data Fusion

**Figure 1:**
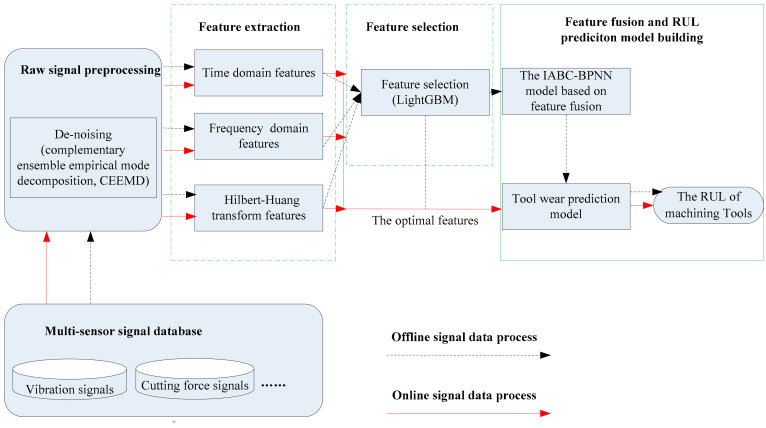
A remaining useful life (RUL) prediction system of machining tools based on multi-sensor data fusion.

As shown in Figure 1, the proposed RUL prediction system of machining tools based on multi-sensor data fusion is consisted of five parts: multi-sensor signal database (offline and online data), signal preprocess(de-noising), feature extraction, feature selection, feature fusion based on the IABC-BPNN model and RUL prediction by PCF method. The system involves two types of signal data process: offline and online. 

For offline signal data process, multi-sensors, such as vibration and force, are installed around the workpiece to acquire different signals from CNC machining tools. First, a large volume of signal data from different sensors that are regularly received and stored in multi-sensor signal database. Next, these stored raw signal data are de-noised by CEEMD and features in time domain, frequency domain and time-frequency domain are extracted. The optimal features, which are those that are more related to tool wear, are selected by LightGBM method from all the extracted features. Finally, the selected features are inputted into the IABC-BPNN model to train and then to predict tool wear.

Once the trained model based on IABC-BPNN is proven to be feasible, it will trigger the process of online signal data process. Multi-sensor online signals are first acquired and de-noised by CEEMD. Next, three types of features are extracted and then are selected. Finally, the selected features as input data are sent to the trained model to obtain the tool wear. According to the tool wear levels, the RUL of machining tools is predicted using PCF.

## 3. Signal Preprocess

Due to the influence of the processing environment and other unavoidable factors, raw signals acquired from multi-sensors contain a lot of redundant information with noise, while the redundant information has a certain interference on the analysis of the signal, and affects the state monitoring of the equipment during the machining process, so further signal preprocess is needed before analysis. 

De-noising is the most common method for signal preprocess. There are many methods for de-noising, amongst which wavelet threshold de-noising and empirical mode decomposition (EMD) are commonly used. The former needs to select the wavelet basis function, the number of decomposition layers, the threshold value, the threshold function, etc., which affect the accuracy of the final de-noising effect; while the latter does not need to set any basis function with prior knowledge, decomposes the signal into a set of intrinsic mode functions (IMFs) and a residue according to the time scale characteristics of the data, and each IMF component decomposed contains the local characteristics of different time scales of the original signals and can efficiently control the level of noise. Therefore, EMD is adaptive and suitable for analyzing non-linear and non-stationary signal sequences. 

However, there are also some problems with EMD, among which is mode mixing problem. To deal with the problem, this paper introduces the CEEMD method proposed by Yeh et al. [24], which is an improved EMD method. The CEEMD method is mainly to add two opposite white noise signals to the analyzed signal many times, then perform EMD decomposition separately, and average the results of the multiple decompositions to obtain the final IMF. With enough the ensemble number of the white noise time series, noise in the signal can be reduced, or even completely eliminated.

**Figure 2:**
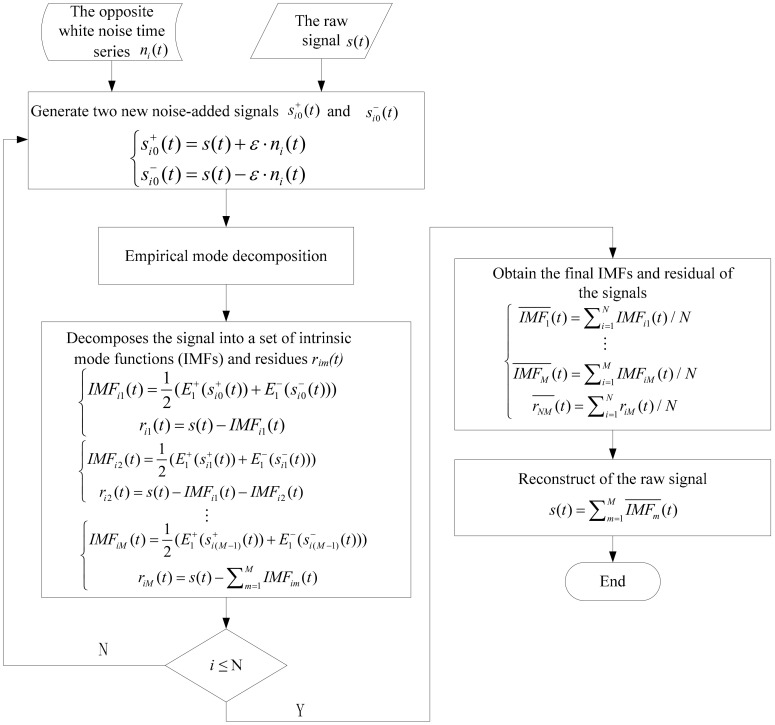
The flow chart of complementary ensemble empirical mode decomposition (CEEMD) preprocess for multi-sensor signals.

Figure 2 shows the flow chart of CEEMD preprocess for multi-sensor signals. The specific steps are described as follows.

(1) The opposite white noise time series $n_{i}(t)$, whose variance is unity and mean value is zero, are added to the raw signal s(t) respectively and two new noise-added signal $s_{i0}^{+}(t)$ and $s_{i0}^{-}(t)$ are produced and expressed as
  (1) $$\left\{\begin{array}{c} s_{i0}^{+}(t)=s(t)+\varepsilon \cdot n_{i}(t)\\ s_{i0}^{-}(t)=s(t)-\varepsilon \cdot n_{i}(t) \end{array}\right.i=1,2,\ldots ,N$$
  where N is the number of ensemble and set to 80, and ε is the signal to noise ratio coefficient and set to [0.1, 0.2].
(2) The two new noised-added signal $s_{i0}^{+}(t)$ and $s_{i0}^{-}(t)$ are discomposed into the first IMF $E_{1}^{+}(s_{i0}^{+}(t))$ and $E_{1}^{-}(s_{i0}^{-}(t))$ using EMD method, then $IMF_{i1}(t)$ can be described as
  (2) $$IMF_{i1}(t)=\frac{1}{2}(E_{1}^{+}(s_{i0}^{+}(t))+E_{1}^{-}(s_{i0}^{-}(t)))$$
  The first residue $r_{i1}(t)$ can be calculated as
  (3) $$r_{i1}(t)=s(t)-IMF_{i1}(t)$$
  If $r_{i1}(t)$ is monotonic, the decomposition will stop. Otherwise, two new noise-added signal $s_{i1}^{+}(t)$ and $s_{i1}^{-}(t)$ are produced by adding the opposite white noise time series $E_{1}(n_{i}(t))$ into $r_{i1}(t)$ and expressed as
  (4) $$\left\{\begin{array}{c} s_{i1}^{+}(t)=r_{i1}(t)+\varepsilon_{1}\cdot E_{1}(n_{i}(t))\\ s_{i1}^{-}(t)=r_{i1}(t)-\varepsilon_{1}\cdot E_{1}(n_{i}(t)) \end{array}\right.$$
  according to the above decomposition process, the second IMF and the second residue $r_{i2}(t)$ are calculated as
  (5) $$IMF_{i2}(t)=\frac{1}{2}(E_{1}^{+}(s_{i1}^{+}(t))+E_{1}^{-}(s_{i1}^{-}(t)))$$
  (6) $$r_{i2}(t)=r_{i1}(t)-IMF_{i2}(t)=s(t)-IMF_{i1}(t)-IMF_{i2}(t)$$
  The above decomposition is repeated until the residue is monotonic, and the final IMF and residue $r_{iM}(t)$ can be given as
  (7) $$IMF_{iM}(t)=\frac{1}{2}(E_{1}^{+}(s_{i(M-1)}^{+}(t))+E_{1}^{-}(s_{i(M-1)}^{-}(t)))$$
  (8) $$r_{iM}(t)=s(t)-\sum_{m=1}^{M}IMF_{im}(t)$$
  where M represents the number of signal decompositions and IMFs, and $r_{iM}(t)$ can be thought of as $IMF_{i(M+1)}(t)$.
(3) Repeating the above two steps for N trials and adding the opposite white noise time series into the signal very trial, we will obtain the final IMFs and residual of the signals, which are expressed as:
  (9) $$\left\{\begin{array}{c} \bar{IMF_{1}}(t)=\sum_{i=1}^{N}IMF_{i1}(t)/N\\ \vdots \\ \bar{IMF_{M}}(t)=\sum_{i=1}^{M}IMF_{iM}(t)/N\\ \bar{r_{NM}}(t)=\sum_{i=1}^{N}r_{iM}(t)/N \end{array}\right.$$
  Finally, the effective IMFs are selected to eliminate the noise in sensor signals, and the reconstruction of the raw signal can be expressed as
  (10) $$s(t)=\sum_{m=1}^{M}\bar{IMF_{m}}(t)$$

## 4. Feature Extraction and Selection

By extracting and analyzing features in time domain (TD), frequency domain (FD) and time-frequency domain (TFD) of the de-noised signals, the evolution of randomness or nonlinearity for machining tools can be tracked and described, so as to establish the RUL of machining tools prediction model.

### 4.1. Feature Extraction of the Multi-Sensor Signals

TD features (TDFs), FD features (FDFs) and TFD features (TFDFs) can reflect the state change of tools during machining, and they are also the effective features for the RUL prediction of machining tools [23,25,26]. By processing the multi-sensor signals after de-noising, TDFs, FDFs and TFDFs of signals at different stages during the machining process are extracted.

In this paper, a total of 10 TDFs are extracted from the multi-sensor de-noising signals by statistical analysis, which include mean value (T_mv_), maximum (T_max_), root mean square (T_rms_), variance (T_vr_), standard deviation (T_sd_), peak-to-peak (T_p2p_), waveform factor (T_wf_), skewness factor (T_sf_), kurtosis factor (T_kf_) and crest factor (T_cf_). Among them, T_mv_, T_max_, T_rms_, T_vr_, T_sd_ and T_p2p_ reflect the amplitude and energy of the signals over time domain, while T_wf_, T_sf_, T_kf_ and T_cf_ reflect the distribution situation over time domain. In frequency domain, a total of 7 FDFs are extracted by power spectrum density analysis, including mean (F_mv_), maximum (F_max_), root mean square (F_rms_), variance (F_vr_), skewness (F_sf_), kurtosis (F_kf_), and relative spectral peak per band (F_rs_) of power spectrum, among which the first five describe the variation of main frequency band position of the signals over frequency domain while the last two describe the dispersion of spectral energies over frequency domain. Table 1 summarizes these TDFs and FDFs, where n is the number of sampling points (in time domain) or spectrum lines (in frequency domain).

In time-frequency domain, TFDFs of the top 10 IMFs of the multi-sensor de-noising signals are extracted by Hilbert-Huang transform (HHT) which is based on the instantaneous frequencies resulting from IMFs of the analyzed signals [27,28,29]. HHT represents a time-frequency domain analysis method of signal by combining EMD with Hilbert transform [30]. Comparing with Fourier spectral analysis and Wavelet packet transform, HHT is mainly based on the instantaneous frequency calculation generated by Hilbert transform of the analyzed signals which are a series of IMFs decomposed by EMD. For any signal s(t), its Hilbert transform H[s(t)] is defined as

(11) $$H[s(t)]=\overset{\wedge }{s}(t)=\frac{1}{\pi }\int_{-\infty }^{\infty }\frac{s(\tau )}{t-\tau }d\tau$$

Then, it can constitute an analytic signal z(t)

(12) $$z(t)=s(t)+j\overset{\wedge }{s}(t)=a(t)e^{j\theta (t)}$$

whose amplitude and instantaneous frequency can be expressed as

(13) $$a(t)=\sqrt{s{\left(t\right)}^{2}+\overset{\wedge }{s}{\left(t\right)}^{2}}$$

(14) $$\omega (t)=\frac{d\theta (t)}{dt}$$

where, $\theta (t)=\arctan[\frac{\overset{\wedge }{s}(t)}{s(t)}]$.

Finally, the Hilbert spectrum of signal energy distribution in time and frequency is denoted as

(15) H(ω,t)=Re(a(t)ej∫ω(t)dt)

where Re denotes the real part of the analytic signal. H(ω,t) reflects the changing law of signal amplitude with time and frequency in the whole frequency band. In this paper, we selected the top 10 IMFs of signal to perform HHT, and any intrinsic energy feature is represented by $E_{k}$:

(16) $$E_{k}=\int {\left(IMF_{K}(t)\right)}^{2}dt\;\;(k=1,\;2,\;\ldots ,\;10)$$

### 4.2. Feature Selection of the Multi-Sensor Signals

Not all of the extracted features are perfectly related to the RUL prediction. On the contrary, some redundant or irrelevant features might reduce the accuracy of the prediction model, thereby decreasing the accuracy and efficiency of online prediction system. Therefore, the optimal feature selection of the multi-sensor signals is very important to improve the performance of the prediction system.

In the paper, the LightGBM method is used to select the optimal features. The literature has confirmed that LightGBM is on the top in machine learning in terms of computational accuracy and running speed, which is especially suitable for the processing of big data [31,32]. LightGBM proposed by Ke et al. [33] is a highly efficient gradient boosting decision tree (GBDT), including two algorithms: gradient-based one-side sampling (GOSS) and exclusive feature bundling (EFB). GOSS is used to split the optimal node in order to acquire a more accurate information gain estimation, while EFB is employed to bundle exclusive features into dense features in order to reduce the size of the training data. Then LightGBM is trained in sequence to fit the negative gradient of loss function in each iteration. According to the weighted combination scheme, LightGBM model $F_{M}(x)$ can be obtained as

(17) $$F_{M}(x)=\sum_{m}^{M}\gamma_{m}h_{m}(x)$$

where m is the iteration number, M is the maximum iteration number, $h_{m}(x)$ represents the base decision tree, x is the data sample, and $\gamma_{m}=\arg\underset{\gamma }{\min\sum_{i=1}^{n}L(y_{i},F_{m})}$ (where n is the total number of features, $L(y_{i},F_{m})$ is the minimum loss function, $F_{m}=F_{m-1}+\gamma_{m}h_{m}(x)$, y is the class label, and x and y combine a training set $\left\{\left(x_{1},y_{1}),(x_{2},y_{2}),\cdots ,(x_{n},y_{n}\right)\right\}$).

The extracted features are input into the LightGBM model for calculation, and the nonlinear relationship between the sequence features (the extracted features) and the class labels (tool wear) is mined. By calling the optimizing function in the encapsulated Sklearn class, the important features will be found in each iteration and given variable importance measures (VIM). The optimal features are selected from these important features with high VIM scores.

In the LightGBM model, VIM usually is expressed using the Gini index (GI) from the random forest (RF) algorithm. Given that there are M features $X_{1},X_{2},\cdots ,X_{c}$, GI score, $VIM_{j}^{\left(Gini\right)}$ of each feature $X_{j}$, is calculated. $VIM_{j}^{\left(Gini\right)}$ represents the average change of node splitting impurity of the jth feature in all RF trees. The formula of GI is

(18) $$GI_{m}=1-\sum_{k=1}^{K}p_{mk}^{2}$$

where K is the number of categories in the sample data set, and $p_{mk}$ is the probability that the sample belongs to category k at node m. The importance of feature $X_{j}$ at node m, this is, GI change before and after node m branching, is

(19) $$VIM_{jm}^{\left(Gini\right)}=GI_{m}-GI_{l}-GI_{r}$$

where $GI_{l}$ and $GI_{r}$ indicate GI of two new nodes after branching, respectively. If the node where feature $X_{j}$ appears in the decision tree *i* is in the set *M*, then the importance of feature $X_{j}$ in the ith tree is

(20) $$VIM_{ij}^{\left(Gini\right)}=\sum_{m\in M}VIM_{jm}^{\left(Gini\right)}$$

Given that there are n trees in RF, then

(21) $$VIM_{j}^{\left(Gini\right)}=\sum_{i=1}^{n}VIM_{ij}^{\left(Gini\right)}$$

Finally, perform a normalization process on all the obtained importance scores to acquire the VIM score of feature $X_{j}$

(22) $$VIM_{j}=VIM_{j}/\sum_{i=1}^{M}VIM_{i}$$

## 5. Feature Extraction and Selection

### 5.1. Feature Fusion and Tool Wear Prediction Model Based on Back Propagation Neural Network Optimized by Improved Artificial Bee Colony (Iabc-Bpnn) Algorithm

Once the optimal features are selected, IABC-BPNN optimization algorithm can be used for feature fusion, and the tool wear prediction model can be trained to obtain tool wear level as a health index of machining tools.

#### 5.1.1. Improved Artificial Bee Colony (IABC) Algorithm

ABC algorithm was proposed and improved by Karaboga et al. [34,35,36], which is a swarm intelligence algorithm and simulates the foraging behaviors of honey bee swarm. The algorithm describes the foraging process of searching the food sources and sharing the information about the found sources among the three groups of bees, including the employed bees, the onlookers and the scouts. The employed bees are connected with the food sources being employed currently, explore the neighborhood through their memory and simultaneously share the information of their food sources with others; the onlookers choose food sources by the information from the employed bees; the role of the scouts is to randomly search a new food source. There is a mutual transformation relationship among them. The employed bees may be transformed into a few onlookers or scouts if they abandon their food sources to search other food sources. The onlookers may be transformed into a few scouts or employed bees if they abandon their food sources and follow other bees to search new ones, or share the information of their food sources with others. The scouts may be transformed into a few employed bees or onlookers if they find new food sources. In the algorithm, the position of a food source represents a candidate solution to a given problem in the search space, and its nectar amount corresponds to the fitness value. The number of the employed bees and the onlookers represents the number of solutions in the population, each of which accounts for half of the population.

Given the number of food sources is SN, the initial population can be represented as $NP=\{X_{1},X_{2},\ldots ,X_{i},\ldots ,X_{SN}\}$ each food source (candidate solution) is represented by $X_{i}=(x_{i1},x_{i2},\ldots ,x_{ij},\ldots ,x_{iN})$ in a N-dimensional search space. In initial stage, the population P is generated by Equation (23)

(23) $$X_{i}^{j}=L^{j}+rand(0,1)(U^{j}-L^{j})$$

where $L^{j}$ and $U^{j}$ are the lower and upper bounds of jth dimension of the search space, respectively.

In the employed bee stage, each employed bee $X_{i}$ will search in its neighborhood to find a new food source (a candidate solution), $new\_X_{i}$, according to Equation (24). Through greedy selection, if the fitness of $new\_X_{i}$ is better than $X_{i}$, then the new one replaces the previous one. When the times of the employed bee search exceeds the threshold limit, the food source is abandoned and a new one is randomly generated.

(24) $$new\_X_{i}^{j}=X_{i}^{j}+R(X_{i}^{j}-X_{k}^{j})$$

where i denotes the current solution, k is a random solution but k≠i, and i,k∈{1,2,…,SN}, j represents the jth element of the corresponding solution, and R is a uniform random number in the rang [−1, 1].

In the onlooker bee stage, the onlooker bee will select a food source according to Equation (25), and this is a way of sharing information between the employed bees and the onlookers. The new solution is updated and selected as in the employed bee stage by Equation (24) and greedy rule.

(25) $$P_{i}=f_{i}/\sum_{m=1}^{M}f_{m}$$

where $P_{i}$ and $f_{i}$ denote the following probability and the fitness of the ith solution, respectively, M is the number of the onlookers in the population, and i∈{1,2,…,M}.

In the scout bee stage, a scout bee searches for new solutions by Equation (23) in the case of the limit is exceeded. The pseudo code of the original ABC algorithm can be described in Algorithm 1.

It is well known that exploration and exploitation are very important for the population-based optimization algorithms, such as GA [37], WOA [38,39] and SSA [40]. In these algorithms, the exploration represents the ability of the algorithm to find the global optimum in the solution space, while the exploitation represents the ability of the algorithm to find a better solution using the previous good solution. In practice, whether an algorithm has good optimization performance mainly depends on whether it can balance the exploration and exploitation abilities well. In the ABC algorithm, the generation of a new candidate solution is based on the change in position (close to or far away) between the current solution and another randomly selected solution in the population by Equation (24). This randomness leads to the new candidate solution is not necessarily better than the previous one. In addition, R is a uniform random number, which also greatly increases the random exploration ability of Equation (24). In summary, the solution search equation described in Equation (24) is more exploratory but insufficiently exploitable.
**Algorithm 1.** The pseudo code of ABC1. **Intialization stage:** Initialize the population **Repeat** 2. **Employed bee stage:** Each employed bee to search new food sources in neighborhood. 3. **Onlooker bee stage:** Each onlooker bee to search new food sources by the probability $P_{i}$. 4. **Scout bee stage:** Each scout bee to search new food sources randomly. 5. **Record the best solution:** Record the best solution found by all current bees. **Until** (stop conditions are met)

In order to improve the exploitation ability of ABC in the process of optimization, many scholars have rewritten Equation (24) in the form of Equation (26), by adding a term called global-best term close to or far away the global optimal solution ($X_{g}$) [41,42,43].

(26) $$new\_X_{i}^{j}=X_{i}^{j}+R(X_{i}^{j}-X_{k}^{j})+\beta (X_{g}^{j}-X_{i}^{j})$$

where β is a uniform number ranged in [0,C], where C is a nonnegative constant. By adjusting the value of β, the exploration and exploitation ability of the algorithm can be well balanced, but the global optimization ability can also be reduced in a certain degree.

In this paper, we improve ABC algorithm by replacing Equation (24) with Equation (27), which combines two search strategies form Equation (24) and Equation (26). In the early stage of the iteration, the algorithm is mainly based on the exploration efficiency, which can quickly find the global optimization, and also has a certain local exploitation ability. In the later stage of the iteration, the algorithm is mainly based on the exploitation ability, which can quickly jump out of the local optimization, and also has a certain global exploration efficiency. The solution search equation is described as

(27) $$new\_X_{i}^{j}=\left\{\begin{array}{c} X_{i}^{j}+R(X_{i}^{j}-X_{k}^{j})\\ X_{i}^{j}+R(X_{i}^{j}-X_{k}^{j})+\alpha (X_{g}^{j}-X_{i}^{j}) \end{array}\right.\begin{array}{c} \begin{array}{ccc} & & rand(0,1)<cr \end{array}\\ \begin{array}{cc} & \;\;\;others \end{array} \end{array}$$

(28) $$\alpha =round(\frac{iter}{iter+L+b*\max iter})*(\frac{1}{iter})$$

where cr= 0.3, α is the variable step coefficient, *b* is an adjustment parameter,iter denotes the number of current iteration,maxiter is the maximum number of iterations, and round() is the rounding function.

**Figure 3:**
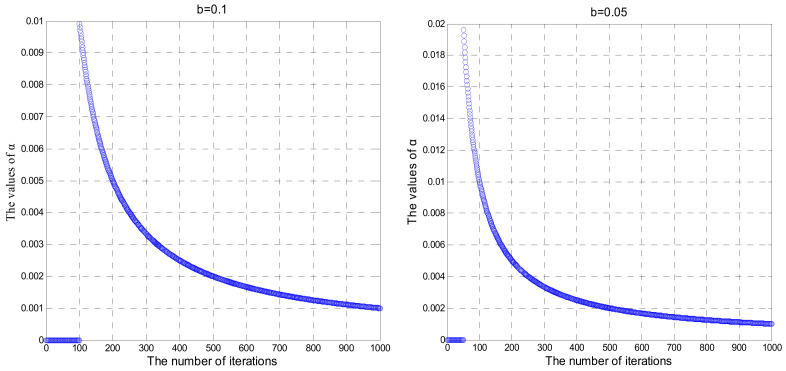
The relationship between b,α and the number of iterations.

As the optimization approaches to the optimal value, the step size in this process should be gradually reduced to decrease the turbulence around the optimal value. The relationship between the variable step coefficient α and the number of iterations is shown in Figure 3. In the process of iteration, the change of α is controlled by adjusting the value of b, which affects the time when the global-best term participates in the iteration. The smaller the value of b, the larger the value range of α is, the earlier the global best term participates in the iteration, and vice versa. The pseudo code of the IABC algorithm is described in Algorithm 2.

**Algorithm 2.** The pseudo code of IABC1. **Intialization stage:** Initialize the population **Repeat** 2. **Employed bee stage:** Each employed bee to search new food sources in neighborhood. New food sources are generated by Equation (27) 3. **Onlooker bee stage:** Each onlooker bee to search new food sources by the probability $P_{i}$. New food sources are generated by Equation (27). 4. **Scout bee stage:** Each scout bee to search new food sources randomly. 5. **Record the best solution:** Record the best solution found by all current bees.**Until** (stop conditions are met)

#### 5.1.2. Back Propagation Neural Network (BPNN)

BPNN is a multi-layer feed-forward neural network using an error back propagation algorithm, which contains an input layer, an output layer, and one or more hidden layers. As a result of its simple structure and being easy to realize, it is widely applied in various aspects, such as prediction and pattern recognition [44,45,46].

**Figure 4:**
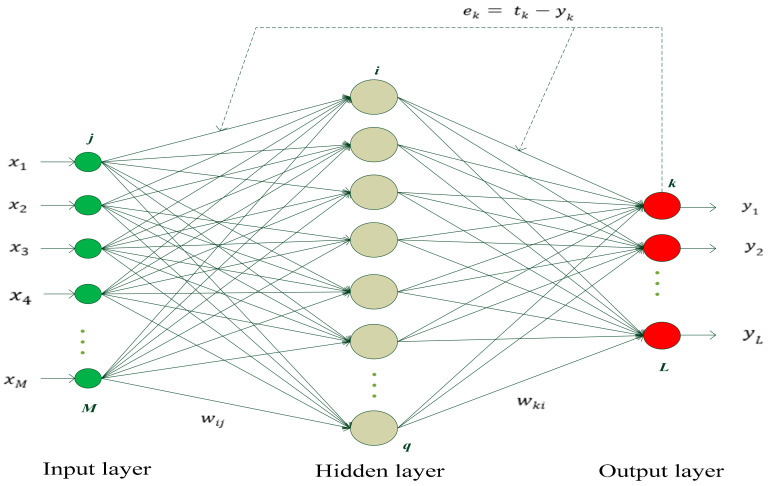
The structure of the back propagation neural network (BPNN).

The structure of BPNN is shown in the Figure 4, where j∈{1,2,…,M},i∈{1,2,…,q}, k∈{1,2,…,L} represent the number of input layer neurons, hidden layer neurons and output layer neurons, respectively; $x_{1},x_{2},\ldots ,x_{M}$, $y_{1},y_{2},\ldots ,y_{L}$ and $t_{k}(k=1,2,\ldots ,L)$ denote the actual input and output, and target output of network, respectively; $e_{k}(k=1,2,\ldots ,L)$ is the output error of the network; $w_{ij}$ and $w_{ki}$ denote the connection weight of between input layer and hidden layer and between hidden layer and output layer, respectively.

The input and output expressions of the hidden layer are expressed, respectively, as

(29) $$HI_{i}=\sum_{j=1}^{M}w_{ij}x_{j}-b_{i}$$

(30) $$HO_{i}=f_{h}(HI_{i})=f_{h}(\sum_{j=1}^{M}w_{ij}x_{j}-b_{i})$$

where $HI_{i}$ and $HO_{i}$ denote the input and output of the hidden layer neuron j, and $b_{i}$ is the corresponding threshold of the neuron j.

The input and output expressions of the output layer are expressed, respectively, as

(31) $$YI_{k}=\sum_{i=1}^{q}w_{ki}*HO_{i}-b_{k}$$

(32) $$YO_{k}=f_{o}(YI_{k})=f_{o}(\sum_{i=1}^{q}w_{ki}*HO_{i}-b_{k})$$

where $YI_{k}$ and $YO_{k}$ denote the input and output of the output layer neuron k, and $b_{k}$ is the corresponding threshold of the neuron k.

The signal is processed step by step from the input layer to the hidden layer until to the output layer, and each layer parameters only effect the next one. If the result of output layer does not meet to anticipant result, the back propagation will be switched by the network. According to the prediction error, the weight and threshold values can be adjusted continuously to make the outcome close to the expected output. The prediction error is usually expressed by minimizing the mean square error (MSE) of the output layer, as shown in Equation (33)

(33) $$MSE=\frac{1}{2}{\sum_{k=1}^{L}\left(t_{k}-y_{k}\right)}^{2}$$

#### 5.1.3. BPNN Optimized by Improved Artificial Bee Colony Algorithm (IABC-BPNN)

**Figure 5:**
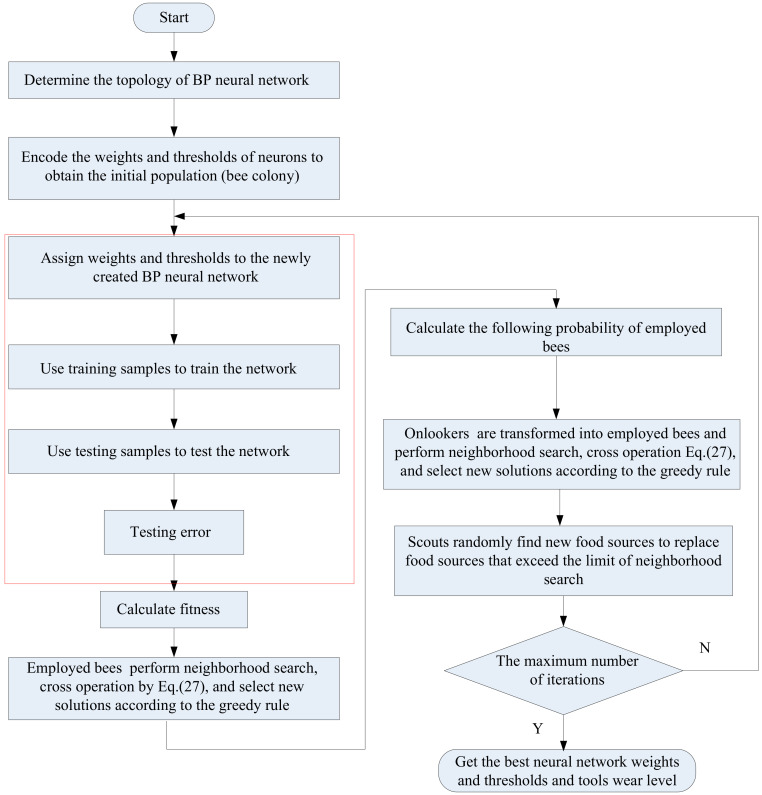
The flowchart of improved artificial bee colony (IABC)-BPNN algorithm.

The BPNN optimized by improved artificial bee colony algorithm (IABC-BPNN) takes the selected features as the input of BPNN, and the weights and thresholds of neurons as a bee individual for ABC algorithm as shown in Figure 5, in which the thresholds and weights of BPNN are optimized by IABC, thus, avoiding falling into local optimization early, and improving the optimization ability of the algorithm.

### 5.2. The Rul Prediction of Machining Tools Base on A Polynomial Curve Fitting

A polynomial curve fitting method is used to fit the tool wear data from the output of IABC-BPNN. The polynomial function is described as follows:

(34) $$f(x_{i})=l_{0}+l_{1}x_{i}+l_{2}x_{i}^{2}+\cdots +l_{n}x_{i}^{n}=\sum_{j=0}^{n}l_{j}x_{i}^{j}$$

where $x_{i}$ is the number of ith machining, $l_{j}$ is the coefficient of the least squares polynomial by computing, and n is a polynomial factorial.

Next, referring to the wear standard of machining tools, the max machining times MT of the machining tool can be deduced by regression analysis of the curve. The RUL of machining tools may be obtained as follows:

(35) $$RUL_{i}=MT-MT_{i}$$

where $MT_{i}$ is the machining times of the ith.

## 6. Experiments and Analysis

### 6.1. Experimental Equipment and Data Description

**Figure 6:**
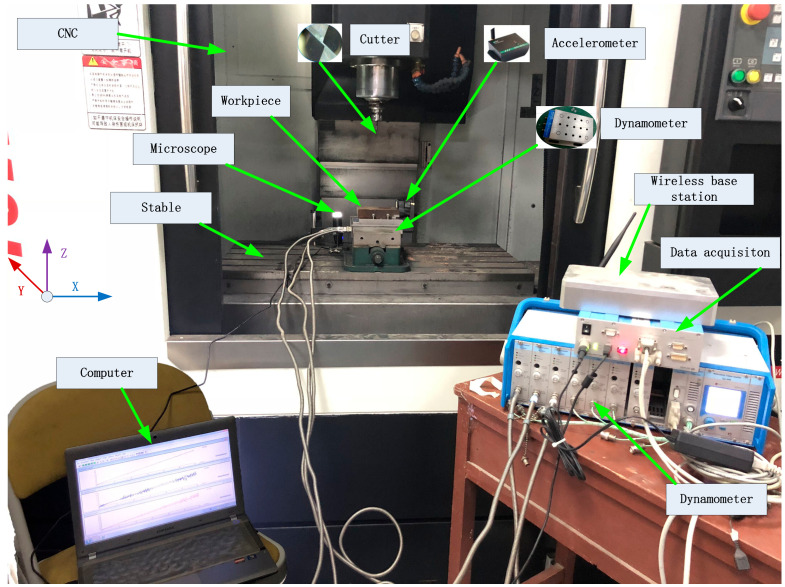
The experimental equipment.

This study uses a CNC milling machine to perform the milling experiment of the tool, and multi-sensors to collect the data generated during the cutting process to verity the effectiveness of the RUL prediction system of machining tools proposed. Figure 6 shows the experimental equipment and connection diagram for measuring tool wear and predicting the RUL. The experimental equipment includes a CNC vertical machining center (G-VM8L, Spindle speed 50–8000 rpm/min, Cutting feed speed X, Y, Z: 5–6000 mm/min), two types of sensors (vibration sensor M69 and force sensor Kistler 9257 A), as well as their supporting charge amplifiers, data acquisitions card and software measuring system, a portable digital microscope (MSUSB401), a notebook, a workpiece (material:C45E, size: 250 mm × 100 mm × 70 mm) and five milling tools (two-edge micro-grain tungsten steel milling cutter SJY H550, type:D6 × 15 × 50 × 2F, HRC 55).

In the G-VM8L CNC center, the workpiece (C45E) is dry milled using a two-edge micro-grain tungsten steel milling cutter with a diameter of 6 mm. The spindle speed is 1200 rpm/min, the milling depth in the z-axis direction is 0.2 mm, the feed rate is 200 mm/min, and the machining length in the feed direction (y-axis) is 70 mm. Each time the machining in the feed direction is completed, the cutter returns to the starting point and is taken a photo with the portable digital microscope MSUSB401 after a pause, and then the next machining operation repeats. The microscope and its own application software can acquire and store images, and measure and record the tool wear after each cut during dry milling operations [23]. In this experiment, the cutter is used to machine the groove of the workpiece, each cutter is machined 300 times or cuts and the total cutting length is 70 mm. There are five milling tools called Ci (i = 1, 2, 3, 4, 5), among which C1 and C3 are measured by the microscope and used as the offline tool wear data to train the prediction model, C2 and C4 are used as the offline data to test the prediction model, and C5 is used as the online data to predict the machining tool RUL.

In the milling process, the signals are simultaneously acquired at 1 KHz sampling frequency by the wireless three-axis Accelerometer M69 (vibration sensor) and Dynamometer Kistler 9257A (force sensor), which are respectively installed on the workpiece, and the between of the workpiece and the table. Specifically, Kistler 9257A is fixed on the worktable, and the workpiece is mounted on the clamping table of Kistler 9257A, which is used to measure the force signal of the workpiece during processing; M69 is fixed on the non-milled surface of the workpiece to measure the vibration signal of the workpiece during processing. M69 collects the cutting vibrations in three directions, whose coordinate system is consistent with the CNC’s as in Figure 6. The cutting vibration signals are sent to a computer after being conditioned by the supporting wireless base station, and displayed in real time by the software MKServer installed on the computer. Simultaneously, Kistler 9257A, as well as its charge amplifier and data acquisition card, collects the cutting forces in three directions, which are also sent to the computer and displayed in real time by the software DynoWare.

### 6.2. Results and Analysis

**Figure 7:**
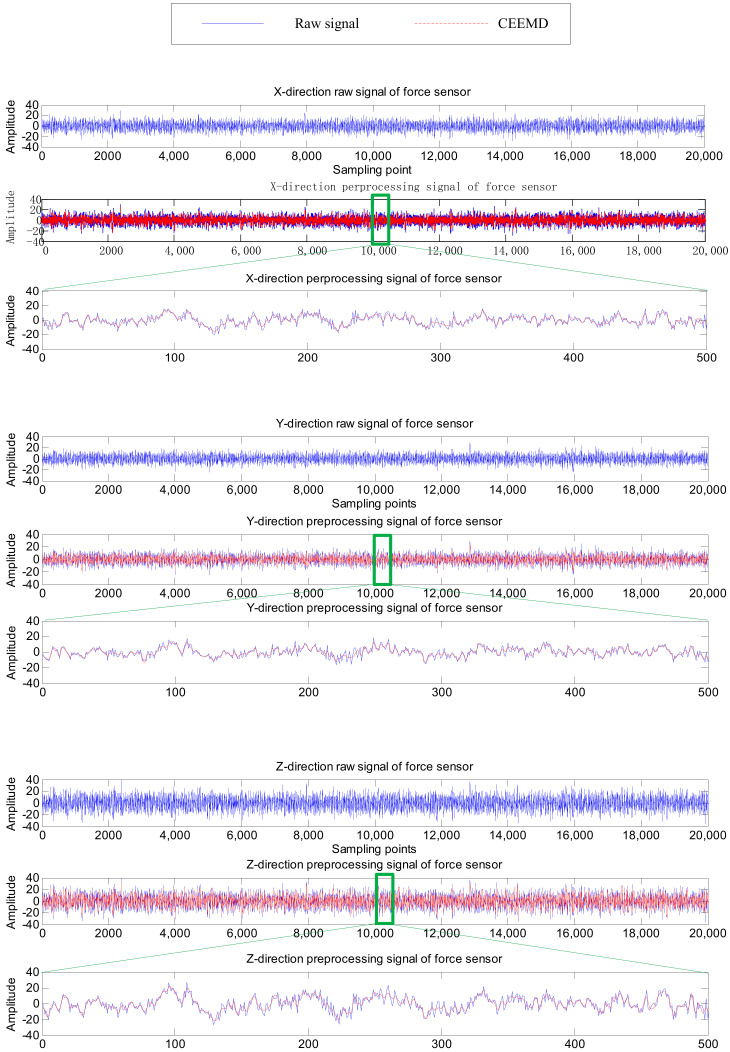
The raw and preprocessed signals of force sensor in one cut.

**Figure 8:**
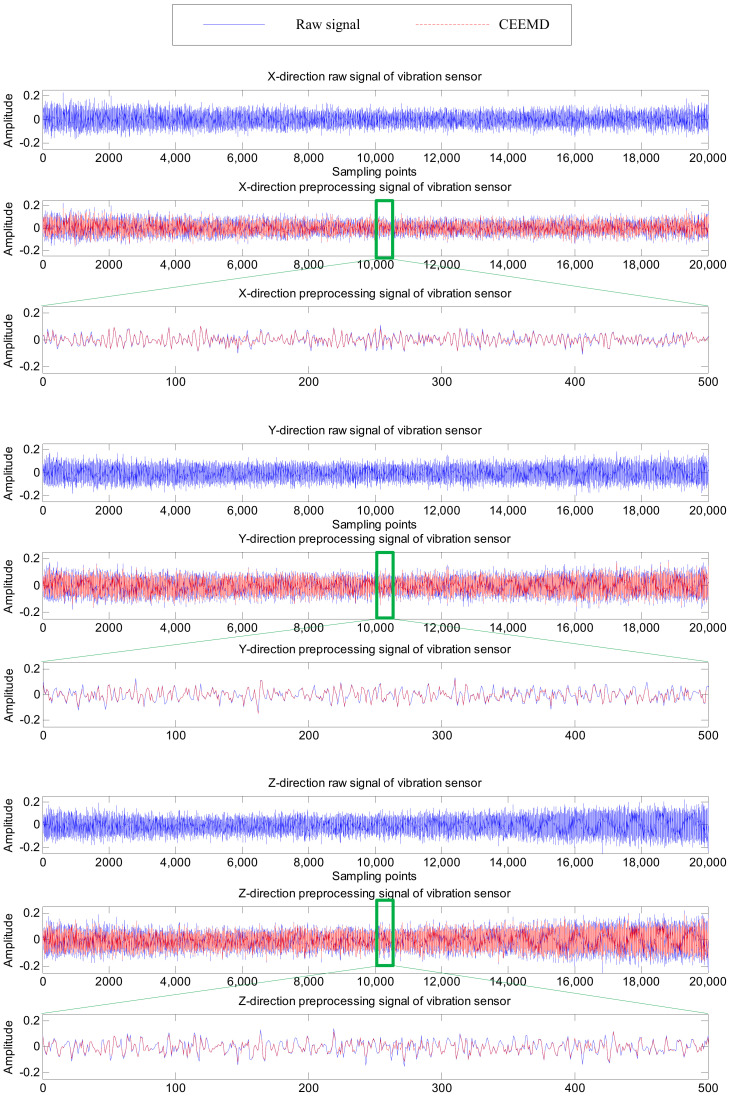
The raw and preprocessed signals of vibration sensor in one cut.

As shown in Figure 7 and Figure 8, six channel signals from the two sensors for one machining process are sampled and preprocessed by CEEMD. Their waveforms in different directions reflect the changes in the force or displacement of the cutter at a certain moment during the machining process, and are also different manifestations of tool wear, which are conducive to more accurately extract features for RUL prediction. The reconstructed signals (marked as CEEMD in Figure 7 and Figure 8) almost coincide with the raw signals, which indicates the CEEMD method can effectively decompose the raw signals.

**Figure 9:**
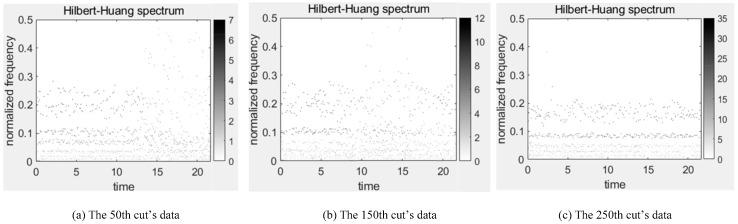
Hibert-Huang spectrum diagrams for the Z-direction of force sensor in C1.

**Figure 10:**
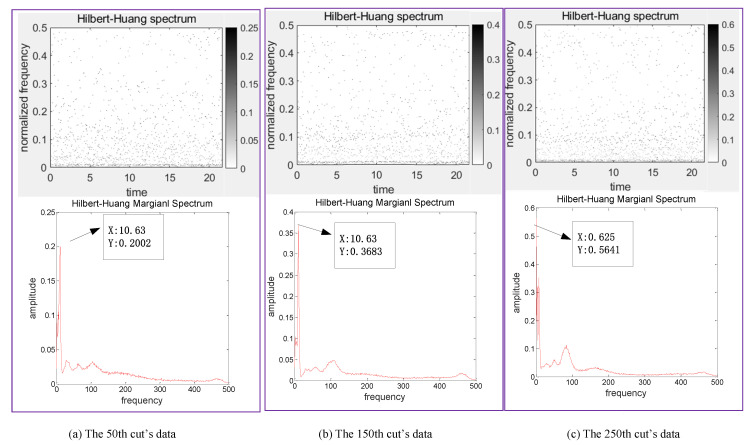
Hibert-Huang spectrum diagrams for the Z-direction of vibration sensor in C1.

In this paper, TDFs, FDFs and TFDFs are extracted by different analysis methods and every channel of sensors can get 10 TDFs, 7 FDFs and top 10 IMFs’ TFDFs, and, finally, a total of 162 features are acquired. These extracted features can reflect the change trend of tool wear during milling. Taking the data of C1 as an example, the corresponding TFDFs of the force sensor and vibration sensor change trends in Z-direction at the 50th, 150th and 250th cut are shown in Figure 9 and Figure 10. With the increase of tool wear, we can find that the maximal amplitude in Z-direction of the two sensors in the C1 are enhanced, while the change trend of dominant frequency is not obvious in Z-direction of the vibration sensor, and gradually decreased in the force sensor.

**Figure 11:**
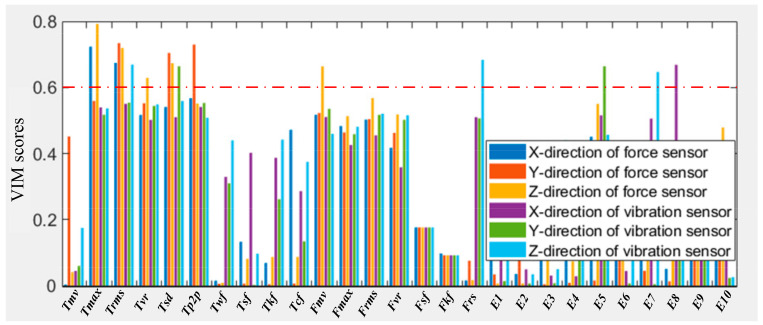
Comparison of variable importance measures (VIM) scores of the all extracted features.

To reduce the dimension of the features and select the optimal features, the lightGBM method is used to carry out the correlation calculation of features and acquire the correlation coefficient scores between different features. High-score features can better reveal the relationship between features and the RUL of the machining tools. From Figure 11, it is found that not all features are suitable to predict the RUL of the machining tools, only those with high VIM scores are selected as the optimal features. For example, 16 optimal features scored greater than 0.6 are selected in total, and they occur in T_max_, T_rms_, T_vr_, T_sd_, T_p2p_, F_mv_, F_vr_, E_5_, E_7_ and E_8_, among which 11 features from the force sensor and six features from the vibration sensor. This shows that the tool wear correlation is related to those features, and, meanwhile, multi-sensor signals can play a very complementary role in predicting the RUL of the machining tools.

Next, the selected 16 features are used as the input of the IABC-BPNN model, and the tool wear as the output. According to the number of the selected features, the model structure of the network can be determined as 16-33-1, which means an input layer with 16 neurons, a hidden layer with 33 neurons and an output layer with 1 neuron. In the IABC-BPNN model, the size of bee colony is 50, the number of employed bees is 25, the dimension of individuals is 595 (528 + 33 + 33 + 1), the learning rate is 0.1, the training target is 0.01, and the max epoch is 500. The IABC-BPNN automatically optimizes the network weights and thresholds, and uses a backward feedback mechanism to train the neural network until the minimal error appears.

**Figure 12:**
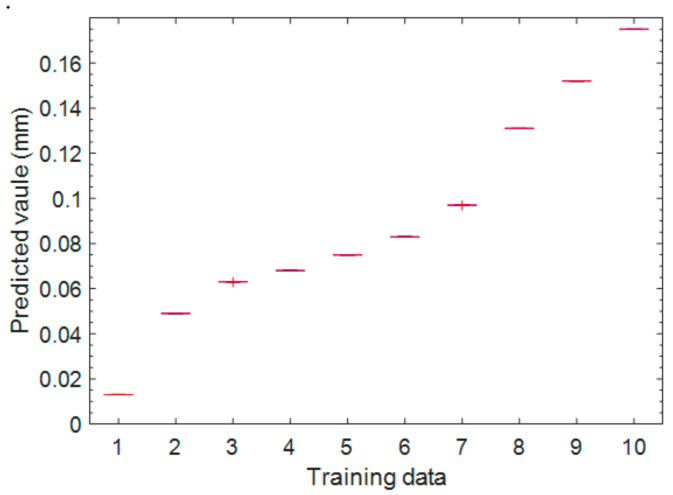
Boxplot comparison of 10 groups of the predicted values.

**Figure 13:**
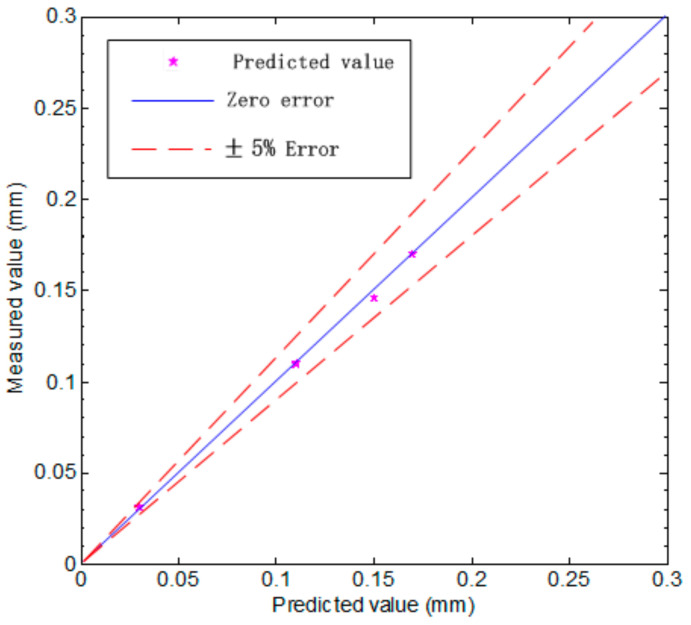
A scatter chart of the predicted value of testing data against the measured value.

After the IABC-BPNN model parameters are determined, the different data with tool wear characteristics in the offline data set from C1 are selected as the training data (10 groups) and the testing data (four groups) from C1, respectively. After the model trained 10 times with 10 groups of training data, the measured values, the predicted values, the predicted values’ standard deviation (STD), error percentage and confidence interval are shown in Table 2, and the results for 10 groups training are depicted Figure 12. Comparing Table 2 and Figure 12, we can easily find that the measured values and predicted values all are in the confidence interval, which are consistent with the results of each training in the box plot, especially the change ranges of the predicted values obtained are very small, indicating the stability of the model is very good. The four groups of testing data are used to test the built model. As shown in Figure 13, the $45^{{}^{\circ}}$ line is a zero-error line, and the predicted value is within 5% error percentage of the $45^{{}^{\circ}}$ line, indicating that the built model is reliable.

**Figure 14:**
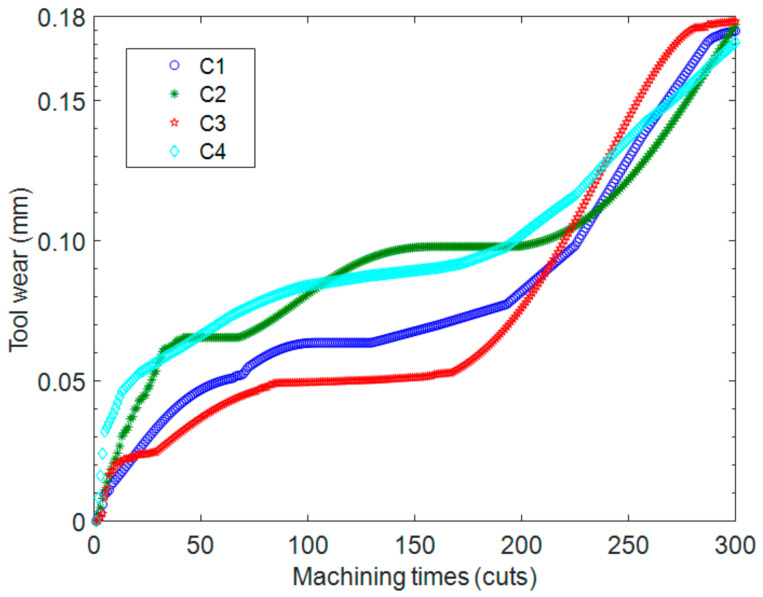
The relationship of tool wear and machining times.

**Figure 15:**
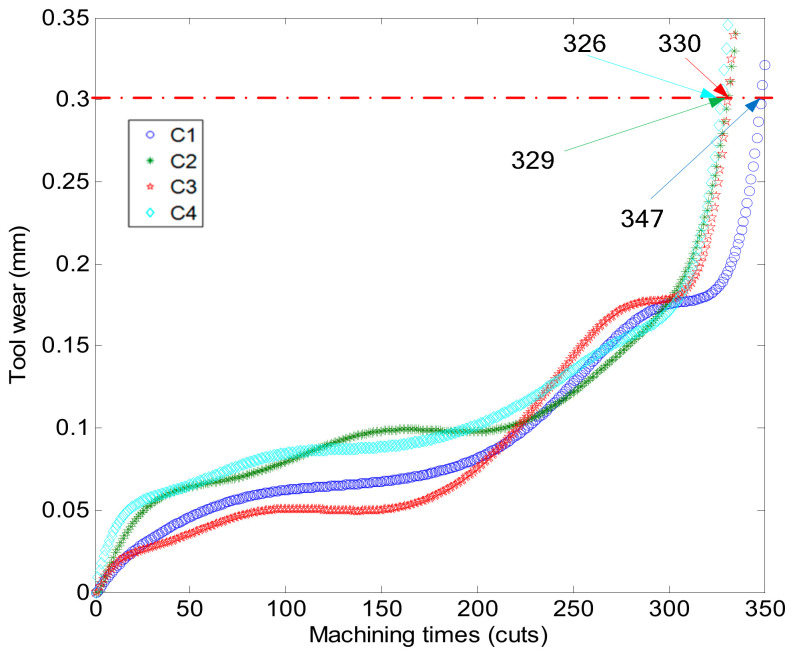
The polynomial curve of the tool wear.

Once the IABC-BPNN model is confirmed, the tool wear values of C2 and C4 can be predicted. Figure 14 describes the relationship between the wear of the 4 tools and the number of machining times, where the wear values of C1 and C3 are measured by the portable digital microscope MSUSB401, while those of C2 and C4 can be predicted by the IABC-BPNN model. According to the PCF method to analyze the data of Figure 14, a polynomial curve of the tool wear can be obtained and descripted in Figure 14. The max tool wear is set to 0.3 mm, and the max machining times MT of the machining tools is computed using regression analysis of the curve, this is, there are 347, 329, 330 and 326 cuts for C1, C2, C3 and C4 respectively. In Figure 15, MT of each tool is represented by the number of corresponding machining times when the polynomial curve of each tool intersects the max tool wear line.

**Figure 16:**
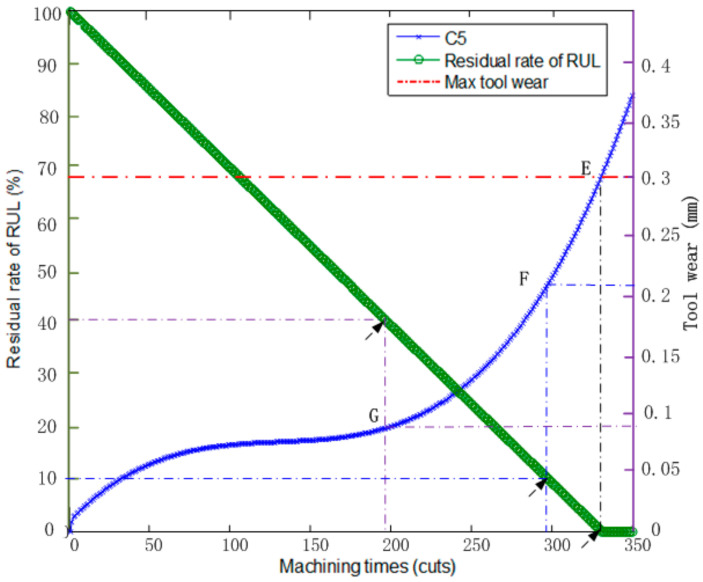
The result of the online RUL prediction.

Then, the established IABC-BPNN can predict the machining tool RUL using the PCF method. After de-noising, feature extraction and selecting the optimal known features, the online data is input into the IABC-BPNN model to predict the tool wear in machining process online. According to Equation (35), the machining tool RUL can be acquired. As shown in Figure 16, the curve of C5 is the tool wear of prediction online, and the residual rate of RUL is expressed by $(\frac{RUL_{i}}{MT})\times 100\%$, so it is easy to find that the RUL corresponding to points E, F and G is 0%, 10% and 40% of MT, respectively. In particular, Point E corresponds to the max wear (0.3 mm) of the machining tool, its corresponding machining times MT = 330, and its corresponding residual rate of RUL is 0%, this is, the RUL of machining tool is zero; point F corresponding to the tool wear is 0.21 mm; its machining times is 297 cuts; its residual rate of RUL is 10%; and the RUL of machining tool is 33 cuts (330 × 10%); point G corresponding to the tool wear is 0.095 mm; its machining times is 198 cuts; its residual rate of RUL is 40%; and the RUL of machining tool is 132 cuts (330 × 40%).

Finally, several compared methods such as NFIS, radial basis function networks (RBFN) and BPNN, are used to predict the tool wear using the same data set (there are 9600 data for C1 and C3). The training target of all these methods is 0.01, the max epoch is 500 and their other parameters are shown in Table 3. Meanwhile, the prediction performances of these methods are measured by such statistical indices as root mean square error (RMSE), mean absolute percentage error (MAPE) and the absolute fraction of the variance (R2). Table 4 gives the results of the statistical performances for the IABC-BPNN and compared methods, from which it can be observed that the proposed method performs better than the others.

## 7. Conclusions and Outlook

In this paper, a novel multi-sensor data fusion for online RUL prediction system of machining tools is proposed. This system integrates multi-sensor signals, signal preprocess, feature extraction, feature selection, IABC-BPNN model-based feature fusion and the RUL prediction, using the PCF method. First, multi-sensor signals from the vibration and force sensors are collected and de-noised by the CEEMD. Then, the multidimensional features are extracted in time domain, frequency domain and time-frequency domain by such methods as statistical analysis, power spectrum density analysis and HHT. Furthermore, the LightGBM method is used to select the optimal features that are important to improve the performance of the prediction system. Next, the IABC-BPNN model-based feature fusion trained by selected features is established and used to implement the tool wear prediction. Finally, an experimental example is implemented to verify the proposed system. The experimental study shows that the proposed method can precisely predict the machining tool RUL which verifies the feasibility of the proposed method in practical application. Meanwhile, compared with its rivals, the IABC-BPNN model shows better prediction and performance.

It should be pointed out that the proposed IABC-BPNN model is only used to predict the tool wear and RUL under a single working condition. In the future, the proposed method will be applied to predict the tool wear and RUL under multi-working conditions and different types of machining tools, and the feasibility of the proposed method is further improved by the automatic optimization of parameters. In addition, the health monitoring of machining tools should be combined with the RUL prediction.

## Figures and Tables

**Table 1:** Features extracted in time domain and frequency domain.

Domain	Feature	Formula
Time	Mean value (Tmv)	$T_{mv}=\sum_{i=1}^{n}x_{i}/n$
	Maximum (Tmax)	$T_{\max}=\max(x_{i})$
	Root mean square (Trms)	$T_{rms}=\sqrt{\sum_{i=1}^{n}x_{i}^{2}/n}$
	Variance (Tvr)	$T_{vr}=\frac{\sum_{i=1}^{n}{\left(x_{i}-T_{mv}\right)}^{2}}{n-1}$
	Standard Deviation (Tsd)	$T_{sd}=\sqrt{T_{vr}}$
	Peak-to-peak (Tp2p)	$T_{p2p}=T_{\max}-\min(x_{i})$
	Waveform Factor (Twf)	$T_{wf}=n\cdot T_{rms}/(\sum_{i}^{n}\left|x_{i}\right|)$
	Skewness Factor (Tsf)	$T_{sf}=\frac{\sum_{i=1}^{n}{\left(x_{i}-T_{mv}\right)}^{3}}{n\cdot T_{sd}^{3}}$
	Kurtosis Factor (Tkf)	$T_{kf}=\frac{\sum_{i=1}^{n}{\left(x_{i}-T_{mv}\right)}^{4}}{n\cdot T_{sd}^{4}}-3$
	Crest Factor (Tcf)	$F_{cf}=\max(\left|x_{i}\right|)/T_{rms}$
Frequency	Mean of power spectrum (Fmv)	$F_{mv}=\sum_{i=1}^{n}S_{i}/n$
	Maximum of power spectrum (Fmax)	$F_{\max}=\max(S_{i})$
	Root mean square of power spectrum (Frms)	$F_{rms}=\sqrt{\sum_{i=1}^{n}S_{i}^{2}/n}$
	Variance of power spectrum (Fvr)	$F_{vr}=\frac{\sum_{i=1}^{n}{\left(S_{i}-F_{mv}\right)}^{2}}{n-1}$
	Skewness of power spectrum (Fsf)	$F_{sf}=\frac{1}{n}\frac{\sum_{i=1}^{n}{\left(S_{i}-F_{mv}\right)}^{3}}{F_{vr}^{3/2}}$
	Kurtosis of power spectrum (Fkf)	$F_{kf}=\frac{1}{n}\frac{\sum_{i=1}^{n}{\left(S_{i}-F_{mv}\right)}^{3}}{F_{vr}^{2}}$
	Relative spectral peak per band (Frs)	$F_{rs}=\frac{F_{\max}}{F_{mv}}$

**Table 2:** Comparison between the measured and the predicted values in C1.

Measured Value (mm)	Predicted Value (mm)	Predicted Value STD	Error Percentage (%)	Confidence Interval (95%)
0.013	0.01298	3.97911 × 10^−5^	0.15	[0.012967, 0.013023]
0.049	0.04899	8.49575 × 10^−5^	0.02	[0.048987, 0.049109]
0.063	0.06301	4.3729 × 10^−5^	0.02	[0.062962, 0.063024]
0.068	0.06799	4.8074 × 10^−5^	0.01	[0.067966, 0.068034]
0.075	0.07495	6.1101 × 10^−5^	0.07	[0.074926, 0.075014]
0.083	0.08296	4.54606 × 10^−5^	0.05	[0.082947, 0.083013]
0.097	0.09698	4.13656 × 10^−5^	0.02	[0.096950, 0.097010]
0.131	0.13100	5.25885 × 10^−5^	0	[0.130943, 0.131019]
0.152	0.15201	2.83039 × 10^−5^	0.01	[0.151987, 0.152027]
0.175	0.17497	4.08792 × 10^−5^	0.02	[0.174957, 0.175015]

**Table 3:** The parameters setting of the compared methods.

Parameters	RBFN	BPNN	IABC-BPNN	NFIS
Learning rate	0.1	0.1	0.1	0.1
Network layers	3	3	3	5
Network structure	16,250,1	16,33,1	16,33,1	16,64,128,128,1
Data set	9600	9600	9600	9600

**Table 4:** The errors of the compared methods.

Error	RBFN	BPNN	IABC-BPNN	NFIS
RMSE	0.1246	0.0679	0.0024	0.0063
MAPE	0.1563	0.0917	0.0032	0.0055
*R* ^2^	0.6326	0.8405	0.9953	0.9152

## References

[1] Liao L., Köttig F. (2016). A hybrid framework combining data-driven and model-based methods for system remaining useful life prediction. Appl. Soft. Comput..

[2] Liu Y., Hu X., Zhang W. (2019). Remaining useful life prediction based on health index similarity. Reliab. Eng. Syst. Safe..

[3] Chen S.-L., Jen Y. (2000). Data fusion neural network for tool condition monitoring in CNC milling machining. Int. J. Mach. Tool. Manu..

[4] Liao L., Köttig F. (2014). Review of Hybrid Prognostics Approaches for Remaining Useful Life Prediction of Engineered Systems, and an Application to Battery Life Prediction. IEEE Trans. Reliab..

[5] Sun H., Cao D., Zhao Z., Kang X. (2018). A Hybrid Approach to Cutting Tool Remaining Useful Life Prediction Based on the Wiener Process. IEEE Trans. Reliab..

[6] Khelif R., Chebel–Morello B., Zerhouni N. (2015). Experience Based Approach for Li–ion Batteries RUL Prediction. IFAC Pap. Online.

[7] Yan J.H., Isobe N., Lee J. (2007). Fuzzy Logic Combined Logistic Regression Methodology for Gas Turbine First-Stage Nozzle Life Prediction. Appl. Mech. Mater..

[8] Baraldi P., Cadini F., Mangili F., Zio E. (2013). Model-based and data-driven prognostics under different available information. Probab. Eng. Mech..

[9] Pálmai Z. (2013). Proposal for a new theoretical model of the cutting tool’s flank wear. Wear.

[10] Mosallam A., Medjaher K., Zerhouni N. (2014). Data-driven prognostic method based on Bayesian approaches for direct remaining useful life prediction. J. Intell. Manuf..

[11] Li W., Liu T. (2019). Time varying and condition adaptive hidden Markov model for tool wear state estimation and remaining useful life prediction in micro-milling. Mech. Syst. Signal. Process..

[12] Rohani Bastami A., Aasi A., Arghand H.A. (2019). Estimation of Remaining Useful Life of Rolling Element Bearings Using Wavelet Packet Decomposition and Artificial Neural Network. Iran. J. Sci. Technol. Trans. Electr. Eng..

[13] Patil M.A., Tagade P., Hariharan K.S., Kolake S.M., Song T., Yeo T., Doo S. (2015). A novel multistage Support Vector Machine based approach for Li ion battery remaining useful life estimation. Appl. Energy.

[14] Razavi S.A., Najafabadi T.A., Mahmoodian A. Remaining Useful Life Estimation Using ANFIS Algorithm: A Data-Driven Approcah for Prognostics. Proceedings of the 2018 Prognostics and System Health Management Conference (PHM-Chongqing).

[15] Kundu P., Darpe A.K., Kulkarni M.S. (2019). Weibull accelerated failure time regression model for remaining useful life prediction of bearing working under multiple operating conditions. Mech. Syst. Sig. Process..

[16] Wu J., Su Y., Cheng Y., Shao X., Deng C., Liu C. (2018). Multi-sensor information fusion for remaining useful life prediction of machining tools by adaptive network based fuzzy inference system. Appl. Soft Comput..

[17] Cheng Y., Zhu H., Hu K., Wu J., Shao X., Wang Y. (2019). Multisensory data-driven health degradation monitoring of machining tools by generalized multiclass support vector machine. IEEE Access.

[18] Tobon-Mejia D.A., Medjaher K., Zerhouni N. (2012). CNC machine tool’s wear diagnostic and prognostic by using dynamic Bayesian networks. Mech. Syst. Sig. Process..

[19] Benkedjouh T., Medjaher K., Zerhouni N., Rechak S. (2013). Health assessment and life prediction of cutting tools based on support vector regression. J. Intell. Manuf..

[20] Gokulachandran J., Mohandas K. (2013). Comparative study of two soft computing techniques for the prediction of remaining useful life of cutting tools. J. Intell. Manuf..

[21] Sun H., Zhang X., Niu W. (2015). In-process cutting tool remaining useful life evaluation based on operational reliability assessment. Int. J. Adv. Manuf. Tech..

[22] Yu J., Liang S., Tang D., Liu H. (2016). A weighted hidden Markov model approach for continuous-state tool wear monitoring and tool life prediction. Int. J. Adv. Manuf. Tech..

[23] Zhang C., Yao X., Zhang J., Jin H. (2016). Tool Condition Monitoring and Remaining Useful Life Prognostic Based on a Wireless Sensor in Dry Milling Operations. Sensors.

[24] Yeh J.-R., Shieh J.-S., Huang N.E. (2010). Complementary ensemble empirical mode decomposition: A novel noise enhanced data analysis method. Adv. Adapt. Data Anal..

[25] Huang Z., Zhu J., Lei J., Li X., Tian F. (2019). Tool wear predicting based on multi-domain feature fusion by deep convolutional neural network in milling operations. J. Intell. Manuf..

[26] Zhou Y., Xue W. (2018). A Multisensor Fusion Method for Tool Condition Monitoring in Milling. Sensors.

[27] Yan R., Gao R.X. (2006). Hilbert-Huang transform-based vibration signal analysis for machine health monitoring. IEEE Trans. Instrum. Meas..

[28] Susanto A., Liu C.-H., Yamada K., Hwang Y.-R., Tanaka R., Sekiya K. (2018). Application of Hilbert–Huang transform for vibration signal analysis in end-milling. Precis. Eng..

[29] Hoseinzadeh M.S., Khadem S.E., Sadooghi M.S. (2019). Modifying the Hilbert-Huang transform using the nonlinear entropy-based features for early fault detection of ball bearings. Appl. Acoust..

[30] Huang N.E., Wu M.L., Qu W.D., Long S.R., Shen S.S.P. (2003). Applications of Hilbert-Huang transform to non-stationary financial time series analysis. Appl. Stoch. Model. Bus..

[31] Li F., Zhang L., Chen B., Gao D.Z., Cheng Y.J., Zhang X.Y., Yang Y.Z., Gao K., Huang Z.W., Peng J. A Light Gradient Boosting Machine for Remainning Useful Life Estimation of Aircraft Engines. Proceedings of the 2018 21st International Conference on Intelligent Transportation Systems.

[32] Chen C., Zhang Q., Ma Q., Yu B. (2019). LightGBM-PPI: Predicting protein-protein interactions through LightGBM with multi-information fusion. Chemometr. Intell. Lab..

[33] Ke G., Meng Q., Finley T., Wang T., Chen W., Ma W., Ye Q., Liu T.-Y. LightGBM: A Highly Efficient Gradient Boosting Decision Tree. Proceedings of the Advances in Neural Information Processing Systems.

[34] Karaboga D. (2005). An Idea Based on Honey Bee Swarm for Numerical Optimization.

[35] Karaboga D., Basturk B. (2007). A powerful and efficient algorithm for numerical function optimization: Artificial bee colony (ABC) algorithm. J. Global. Optim..

[36] Karaboga D., Basturk B. (2008). On the performance of artificial bee colony (ABC) algorithm. Appl. Soft Comput..

[37] Tang K.S., Man K.F., Kwong S., He Q. (1996). Genetic algorithms and their applications. IEEE Signal. Proc. Mag..

[38] Mirjalili S., Lewis A. (2016). The Whale Optimization Algorithm. Adv. Eng. Softw..

[39] Liu M., Yao X., Li Y. (2020). Hybrid whale optimization algorithm enhanced with Lévy flight and differential evolution for job shop scheduling problems. Appl. Soft Comput..

[40] Jain M., Singh V., Rani A. (2019). A novel nature-inspired algorithm for optimization: Squirrel search algorithm. Swarm Evol. Comput..

[41] Zhu G., Kwong S. (2010). Gbest-guided artificial bee colony algorithm for numerical function optimization. Appl. Math. Comput..

[42] Gao W.-F., Huang L.-L., Wang J., Liu S.-Y., Qin C.-D. (2016). Enhanced artificial bee colony algorithm through differential evolution. Appl. Soft Comput..

[43] Xue Y., Jiang J., Zhao B., Ma T. (2017). A self-adaptive artificial bee colony algorithm based on global best for global optimization. Soft Comput..

[44] Wang D., Luo H., Grunder O., Lin Y., Guo H. (2017). Multi-step ahead electricity price forecasting using a hybrid model based on two-layer decomposition technique and BP neural network optimized by firefly algorithm. Appl. Energy.

[45] Qu Z., Mao W., Zhang K., Zhang W., Li Z. (2019). Multi-step wind speed forecasting based on a hybrid decomposition technique and an improved back-propagation neural network. Renew. Energ..

[46] Jian B.-L., Chang-Jian C.-W., Guo Y.-S., Yu K.-T., Yau H.-T. (2020). Optimizing Back Propagation Neural Network Parameters to Judge Fault Types of Ball Bearings. Sens. Mater..

